# Concerted BAG3 and SIRPα blockade impairs pancreatic tumor growth

**DOI:** 10.1038/s41420-022-00817-9

**Published:** 2022-03-03

**Authors:** Margot De Marco, Vanessa Gauttier, Sabrina Pengam, Caroline Mary, Bianca Ranieri, Anna Basile, Michela Festa, Antonia Falco, Francesca Reppucci, Anna Lisa Cammarota, Fausto Acernese, Vincenzo De Laurenzi, Gianluca Sala, Sergio Brongo, Masayuki Miyasaka, Shabnam Shalapour, Bernard Vanhove, Nicolas Poirier, Roberta Iaccarino, Michael Karin, Maria Caterina Turco, Alessandra Rosati, Liberato Marzullo

**Affiliations:** 1 Department of Medicine, Surgery and Dentistry “Schola Medica Salernitana”, University of Salerno, Baronissi, SA 84081 Italy; 2 BIOUNIVERSA s.r.l., R&D Division, Baronissi, SA 84081 Italy; 3 OSE Immunotherapeutics, Nantes, France; 4 Department of Pharmacy, University of Salerno, Fisciano, SA 84084 Italy; 5 Department of Medical, Oral and Biotechnological Sciences, University “G. d’Annunzio” of Chieti-Pescara, 66100 Chieti, Italy; 6 Immunology Frontier Research Center, Osaka University, Yamada-oka, Suita Japan; 7 Department of Cancer Biology, University of Texas MD Anderson Cancer Center, Houston, TX 77054 USA; 8 Department of Pharmacology, University of California San Diego School of Medicine, La Jolla, CA USA

**Keywords:** Pancreatic cancer, Antibody therapy, Cancer microenvironment

## Abstract

The BAG3- and SIRPα- mediated pathways trigger distinct cellular targets and signaling mechanisms in pancreatic cancer microenvironment. To explore their functional connection, we investigated the effects of their combined blockade on cancer growth in orthotopic allografts of pancreatic cancer mt4–2D cells in immunocompetent mice. The anti-BAG3 + anti-SIRPα mAbs treatment inhibited (*p* = 0.007) tumor growth by about the 70%; also the number of metastatic lesions was decreased, mostly by the effect of the anti-BAG3 mAb. Fibrosis and the expression of the CAF activation marker α-SMA were reduced by about the 30% in animals treated with anti-BAG3 mAb compared to untreated animals, and appeared unaffected by treatment with the anti-SIRPα mAb alone; however, the addition of anti-SIRPα to anti-BAG3 mAb in the combined treatment resulted in a > 60% (*p* < 0.0001) reduction of the fibrotic area and a 70% (*p* < 0.0001) inhibition of CAF α-SMA positivity. Dendritic cells (DCs) and CD8+ lymphocytes, hardly detectable in the tumors of untreated animals, were modestly increased by single treatments, while were much more clearly observable (*p* < 0.0001) in the tumors of the animals subjected to the combined treatment. The effects of BAG3 and SIRPα blockade do not simply reflect the sum of the effects of the single blockades, indicating that the two pathways are connected by regulatory interactions and suggesting, as a proof of principle, the potential therapeutic efficacy of a combined BAG3 and SIRPα blockade in pancreatic cancer.

## Introduction

Pancreatic ductal adenocarcinoma (PDAC) is a lethal malignancy with increasing incidence and mortality trends in several countries [1]. Its responsiveness to therapies, including single-agent immune modulators, is very poor [2–5]. A major role in PDAC resistance to therapy is ascribed to the tumor microenvironment, characterized by extensive desmoplasia, active immunosuppressive pathways, and the contribution of pro-tumor cytokines secreted by tumor-associated macrophages (TAMs), other immune cells, and cancer-associated fibroblasts (CAFs) [6–8]. A combined inhibition of distinct immunosuppressive and/or pro-tumor pathways could represent a strategy capable of circumventing the blocks that affect therapy attempts [2–4, 7].

In the pancreatic cancer microenvironment, two distinct mechanisms involved in supporting tumor growth and suppressing the anti-tumor immune response are mediated by BAG3/BAG3R [9–15] and SIRPα/CD47 [16–24] axes. These two pathways operate in different cell types and through distinct signaling pathways. BAG (*Bcl*-*2*-associated AthanoGene) 3 protein plays a dual role in cancer biology and in resistance to therapy [15]. Indeed, in neoplastic cell cytosol it regulates autophagy [25] and interferes with the Hsp70-mediated delivery of IKKγ [9] and other anti-apoptotic proteins [15] to proteasome, sustaining their levels and cell survival, while, being secreted by pancreatic cancer cells, it binds to a specific receptor (BAG3R) on TAMs, triggering the p38- and Akt-dependent release of pro-tumorigenic cytokines and chemokines [10, 11, 15]. In several pancreatic cancer murine models, BAG3 blockade by a monoclonal antibody impairs the activation of TAMs [11, 12] and CAFs [13]. This effect produces a significant reduction of the tumor growth of both MIA PaCa-2 and patient-derived pancreatic cancer xenografts in immunodeficient mice [11]. Notably, in heterotopic allografts of murine pancreatic cancer cells in immunocompetent syngeneic mice, treatment with the anti-BAG3 mAb sensitizes the tumors to the effect of an anti-PD-1 antibody [12]. On the other hand, signal-regulatory protein (SIRP)α (CD172a or SHPS-1), expressed on myeloid cells, upon its binding to neoplastic cell surface CD47 antigen (“*don’t eat me*” signal) transduces, through its interaction with Src Homology region 2 domain-containing Phosphatases (SHPs), an inhibitory signal, that blocks cancer cell phagocytosis by macrophages and dendritic cell (DC) activation [16–18, 22]. Due to the roles played by DCs and macrophages in antigen presentation and in the release of cytokines that activate cytotoxic cells, the SIRPα/CD47 pathway regulates not only the innate immune activity, but also the adaptive response. Indeed, the blockade of the SIRPα/CD47 pathway reportedly potentiates T cell recruitment into tumor nest and antitumor immune activity in some tumor types [19–21, 23, 24].

The regulatory connections between the BAG3/BAG3R and the SIRPα/CD47 pathways have not yet been explored. We aimed to verify the possible functional interaction between the two mechanisms in regulating pancreatic carcinoma interplay with its microenvironment, by investigating whether their concerted blockade could produce enhanced reductive effects on pancreatic tumor growth and metastatic diffusion.

For this purpose, we studied the effects of an anti-BAG3 [12] and an anti-SIRPα [26] antibody, separately or in combination, in a murine model of pancreatic cancer orthotopic allografts in syngeneic immunocompetent animals.

## Materials and methods

### Animal experiments

The research protocol of the animal study was approved by the Ethics Committee in accordance with the institutional guidelines of the Italian Ministry of Health, protocol n. 407/2019-PR. Female C57BL/6J (6 weeks old; Charles River, Italy) mice were housed five per cage with food and water available ad libitum and maintained on a 12 h light/dark cycle under standard and specific pathogen-free conditions. A total of 48 mice were used and maintained in a barrier facility on HEPA-filtered racks. The number of mice was calculated with the G*power 3 software to obtain a power of 85%, with an α error of 0.05. Suffering mice and those in which the tumor was undetectable were excluded from the experiment. All experiments were conducted in a biological laminar flow hood, and all surgical procedures were conducted with strict adherence to aseptic techniques. The mice were anesthetized using isoflurane. For injecting cancer cells, mice were prepped with 10% povidone-iodine; a longitudinal median laparotomy with a xipho-pubic incision was made, and the tail of the pancreas exteriorized gently. mt4–2D murine pancreatic cancer cells [12, 27] were suspended in 40 µl of PBS 1× in a 1 ml syringe; using a 25G needle, cells were injected into the tail of the pancreas and the injection point dubbed with sterile cotton. Once hemostasis was confirmed, the tail of the pancreas was returned into the abdomen and the wound was closed as a single layer using interrupted 5.0 silk sutures and skin staples. Two weeks after cell injection, tumor area was assessed using Vevo 2100 (Visualsonics, Canada) under anesthesia. Mice randomization into four arms consisting of 12 mice each, was carried out to homogenize the average area (approximately 4 mm^2^) of tumors in each group. Three times per week, one group of animals received i.p. injection of anti-BAG3 [12] (20 mg kg^−1^); another group received i.p. injection of anti-SIRPα (MY1 mIgG1 clone) [26] (10 mg kg^−1^) twice a week; a third group received treatment with both anti-BAG3 and anti-SIRPα antibodies; the control group received i.p. injection of an unrelated IgG (Bioxcell Clone: MOPC-21 Catalog#: BE0083, 20 mg kg^−1^). After two weeks of treatment, the animals were sacrificed, and tumors excised for analysis. The lot of anti-BAG3 mAb produced in CHO were tested for TGFβ1 content [28] and showed a concentration of cytokine level of 45.2 pg per μg of antibodies, corresponding to a calculated amount of 18.1 ng of TGFβ1 co-injected per i.p. administration, per mouse.

### NanoString transcriptional technology analysis

RNA from mouse tumor tissues was extracted by a Trizol-chloroform gradient and isolation with RNeasy Mini kit (Qiagen). Gene expression was quantified by the NanoString nCounter platform, using 50 ng of total RNA for tumor tissue and the Mouse PanCancer Immune Profiling (PCIP) Panel (NanoString Technologies). The code set was hybridized with the RNA overnight at 65 °C; then RNA transcripts were immobilized and counted using the NanoString nCounter Sprint. Normalized expression data were analyzed by using the nSolver software. Lists of genes extracted from heatmaps were tested for their protein interactions using the STRING online software (https://string-db.org/).

### Immunofluorescence

For paraffin-embedded sections, immunofluorescence protocol included deparaffination in Clear-Rite™ 3 (ThermoScientific, Waltham, MA), rehydration through descending degrees of alcohol up to water, non-enzymatic antigen retrieval in sodium citrate buffer 10 mM, 0.05% Tween, pH 6.0, for 40 min in pressure cooker at 95 °C. After washing, non-specific binding was blocked with 10% normal goat serum (NGS) in PBS 1× 1 h, RT. Sections were then incubated with anti-CD8 monoclonal antibody (C8/144B, Thermo Fisher 1:25), anti-CD11c monoclonal antibody (ab33483, Abcam, at 1:25), anti-CD103 monoclonal antibody (DM3536P, OriGene Technologies, at 1:25), anti-α-SMA antibody (A2547, Sigma-Aldrich, at 1:350) overnight at 4 °C in a humidified chamber. After another washing step, sections were incubated with the secondary antibodies (used at 1:200 dilution). Nuclei were counterstained with 1 µg/ml Hoechst 33342 (Molecular Probes, Oregon). Negative controls were performed using all reagents except the primary antibody. Slides were then coverslipped using an aqueous mounting medium and analyzed using a confocal laser scanning microscope (Leica SP5, Leica Microsystems, Wetzlar, Germany). Images were acquired in sequential scan mode by using the same acquisitions parameters (laser intensities, gain photomultipliers, pinhole aperture, ×40 objective) when comparing experimental and control material. For figures preparation, brightness and contrast of images were adjusted by taking care to leave a light cellular fluorescence background, for visual appreciation of the lowest fluorescence intensity features and to help comparison among the different experimental groups. Leica Confocal Software and ImageJ were used for data analysis.

### Picrosirius red staining

Tumors were embedded into paraffin and sections (5 μm), mounted on glass slides, processed, and stained with Picrosirius red (cat. 24901, Polysciences, Inc.) according to the manufacturer’s instructions. At least three different image fields were acquired at 20× magnification. The areas of collagen staining were quantitatively evaluated with ImageJ software and expressed as percentages of the total corresponding area.

### Statistical analysis

Results are shown as standard error of the means (SEM). All statistical analyses were performed with MATLAB R2020b (Mathworks) and GraphPad Prism 8.0.1 (GraphPad Software). A *p* value <0.05 was considered statistically significant and the confidence interval was calculated at 95%. Lilliefors’ composite goodness-of-fit test for normality was used to test the null hypothesis that data came from a normally distributed population. To evaluate the effects of two treatments (anti-BAG3 and anti-SIRPα) on tumor weight and number of metastases, two factor analysis was performed using two-way ANOVA (unbalanced Type III sum of squares). To complete the two-factor analysis, a post hoc comparison (HSD Tukey–Kramer) was conducted and the differences between means of each group with their respective 95% confidence intervals were reported, to estimate the effect size. To assess whether there was a statistically significant effect of treatment on the number of subjects with metastases, a Fisher’s exact test was conducted. Groups were formed for homogeneity of treatment and compared to assess whether and which of the factors had an effect. The effect size was estimated by calculating the Risk Ratio with its confidence intervals in the presence of either or both treatments. For all the other data analyzed, D’Agostino–Pearson test was performed to verify the normal distribution of linear variables. For variables normally distributed, we used one-way ANOVA followed by Bonferroni multiple comparisons test; for variables non-normally distributed, *p* values were evaluated by a non-parametric Kruskal–Wallis matched pairs test with Dunn’s comparison.

## Results

### The combined blockade of BAG3/BAG3R and SIRPα/CD47 pathways decreases tumor growth and the metastatic process

**Fig. 1:**
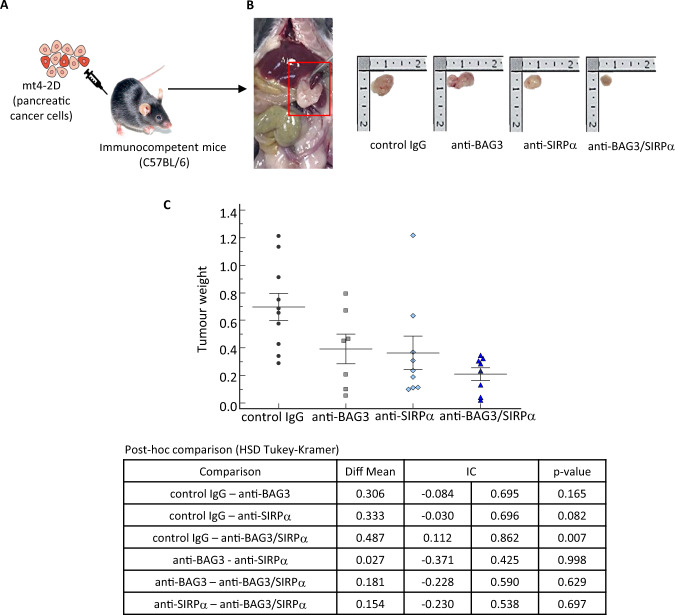
Effect of treatment with anti-SIRPα and anti-BAG3 antibodies on pancreatic cancer growth. **A** mt4–2D cells were injected into the pancreata of 6-week-old C57BL/6J mice. After 15 days tumor area was measured by ultrasound imaging and mice were randomized into four arms consisting of 12 mice each, in which tumor area average was approximately 4 mm^2^. One group received i.p. injection of anti-BAG37 (20 mg kg^−1^) times a week; another group received i.p. injection of anti-SIRPα (10 mg kg^−1^) twice a week; a third group received treatment with both anti-BAG3 and anti-SIRPα antibodies; the control group received i.p. injection of an unrelated IgG (Bioxcell Clone: MOPC-21 Catalog#: BE0083, 20 mg kg^−1^) 3 times a week. Animals were sacrificed when the tumor area measured by ultrasound reached 60 mm^2^. **B** Comparison of representative tumors from the four different groups. **C** Weights of tumors excised from animals treated with control IgG, anti-BAG3 mAb, anti- SIRPα mAb, or both mAbs for two weeks, as described in the “Materials and Methods” section. The mean and individual values in each group are shown (control IgG: *n* = 10; anti-BAG3 mAb: *n* = 7; anti-SIRPα mAb: *n* = 9; anti-BAG3 + anti-SIRPα mAbs: *n* = 8). Two-way ANOVA followed by Tukey–Kramer’s *post hoc* test was used for data analysis.

To verify the potential cooperation of BAG3/BAG3R- and SIRPα/CD47-blockades in impairing pancreatic tumor growth, we produced murine orthotopic pancreatic cancer allografts by injecting murine pancreatic cancer cells (mt4–2D) [12, 27] into the pancreata of syngeneic C57BL6 mice (Fig. 1A) and analyzed the effects of a treatment with anti-BAG3 [12] and anti-SIRPα [26] murine monoclonal antibodies on tumor growth. Mice were sacrificed and tumors excised after two weeks of treatment (Fig. 1B).

**Fig. 2:**
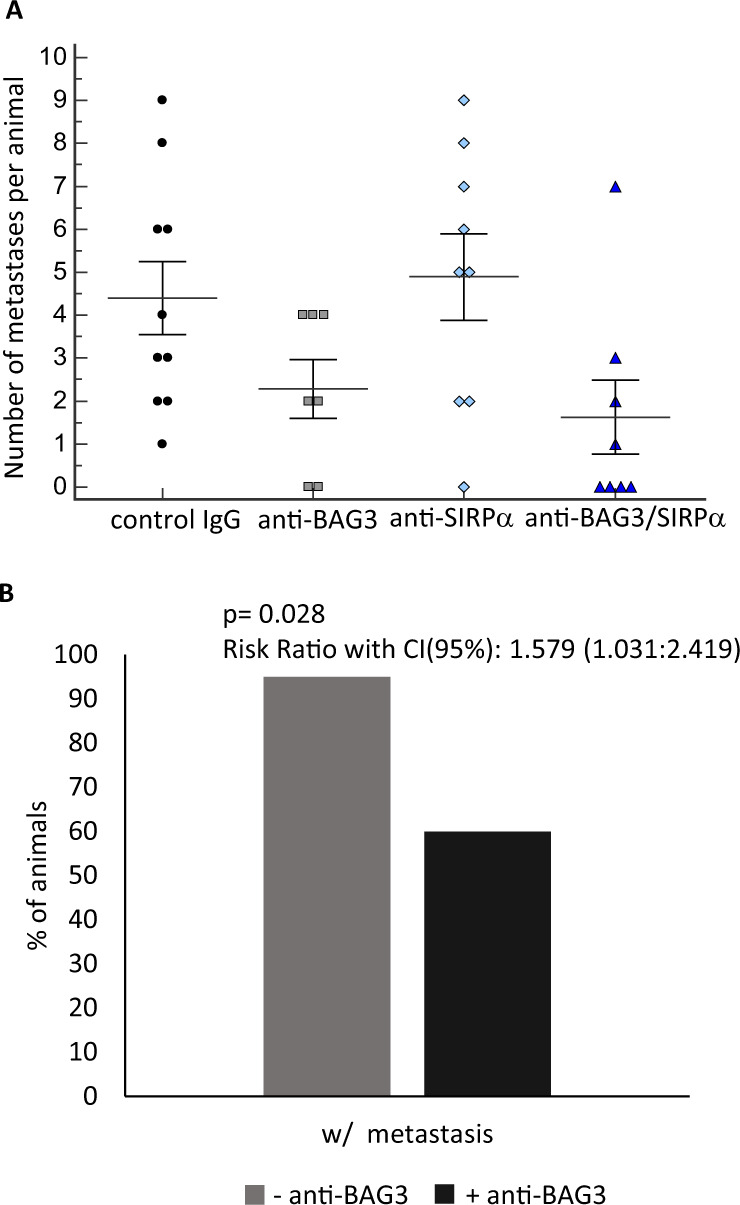
Effect of treatment with anti-SIRPα and anti-BAG3 antibodies on metastatic spreading. **A** Number of metastatic lesions per animal in the four different groups. **B** Considering the major effect of BAG3 single treatment on metastasis (**A**), the histogram represents the overall reduction of metastatic lesions in animal treated with anti-BAG3 mAb. A Fisher’s exact test was conducted to estimate the effect size of combo treatment by calculating the risk ratio with its confidence intervals in the presence or absence of the anti-BAG3 antibody.

In the ex vivo analysis, we found that the treatment with either anti-BAG3 or anti-SIRPα mAb resulted in a reduction of tumor weight, which was more impressive when the two antibodies were used in combination (Fig. 1C). Furthermore, the combined treatment resulted also in a decrease of the number of metastases per animal; in this respect, the effect of the anti-BAG3 antibody appeared to predominate over that of the anti-SIRPα antibody (Fig. 2A, B).

### Expression of genes associated with immunity in treated tumors

**Fig. 3:**
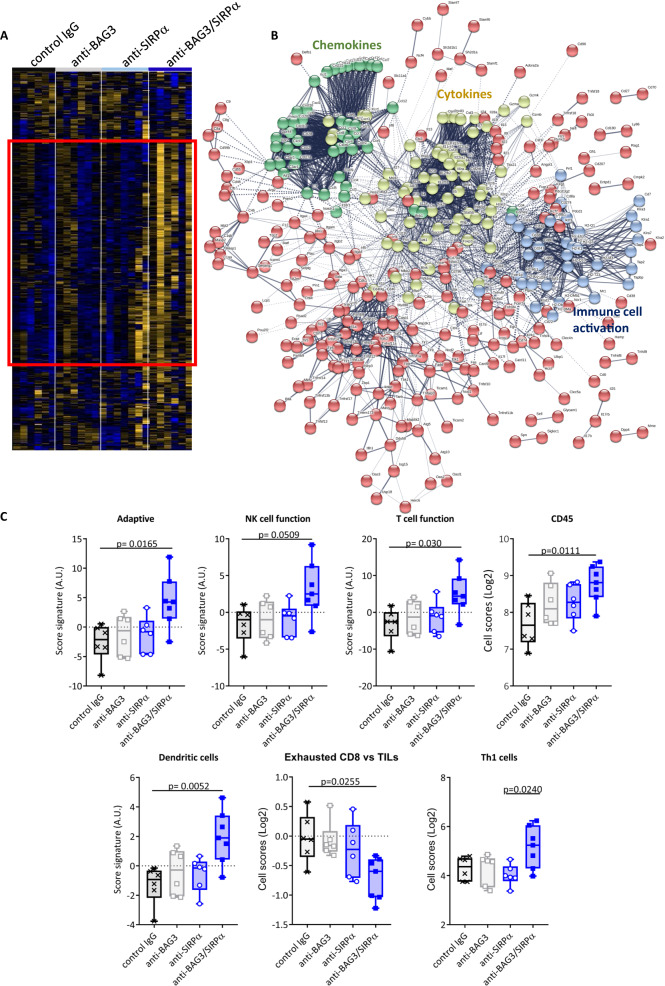
Differential gene expression analysis in tumors from the four treatment groups. **A** Heatmap of the expression of selected genes in tumors excised from animals treated with control IgG, anti-BAG3 mAb, anti- SIRPα mAb, or both mAbs. The heatmap represents median-centered and colorized expression values. **B** STRING protein-protein network analysis of the upregulated gene cluster surrounded by the solid line rectangle in (**A**). **C** Immune cell signature enrichment scores using NanoString transcriptional analysis of excised tumors. One-way ANOVA followed by Bonferroni’s *post hoc* test was used for data analysis.

To investigate the effects of the treatments with the antibodies on the anti-tumor immune response, we analyzed the expression of genes involved in immune functions in tumor tissues, by using a digital multiplexed gene expression platform. As shown in Fig. 3A, slight differences in the expression of these genes were detectable in the anti-SIRPα- or anti-BAG3- treated groups compared to the controls. On the other hand, the tumors from mice treated with both antibodies showed a very different pattern (Fig. 3A). Indeed, in this group, the expression of genes for cytokines or chemokines, and of other genes associated with immune activation, was significantly increased and involved almost entirely the gene family clusters (Fig. 3B). Particularly, we observed an enhanced expression of genes associated with adaptive immunity, such as genes expressed in tumor-infiltrating lymphocytes (TILs), DCs, and T helper (Th) 1 cells, and a consistent down-modulation of exhausted lymphocytes signature (Fig. 3C).

### Infiltrating dendritic cells and CD8^+^ lymphocytes in tumor tissues

**Fig. 4:**
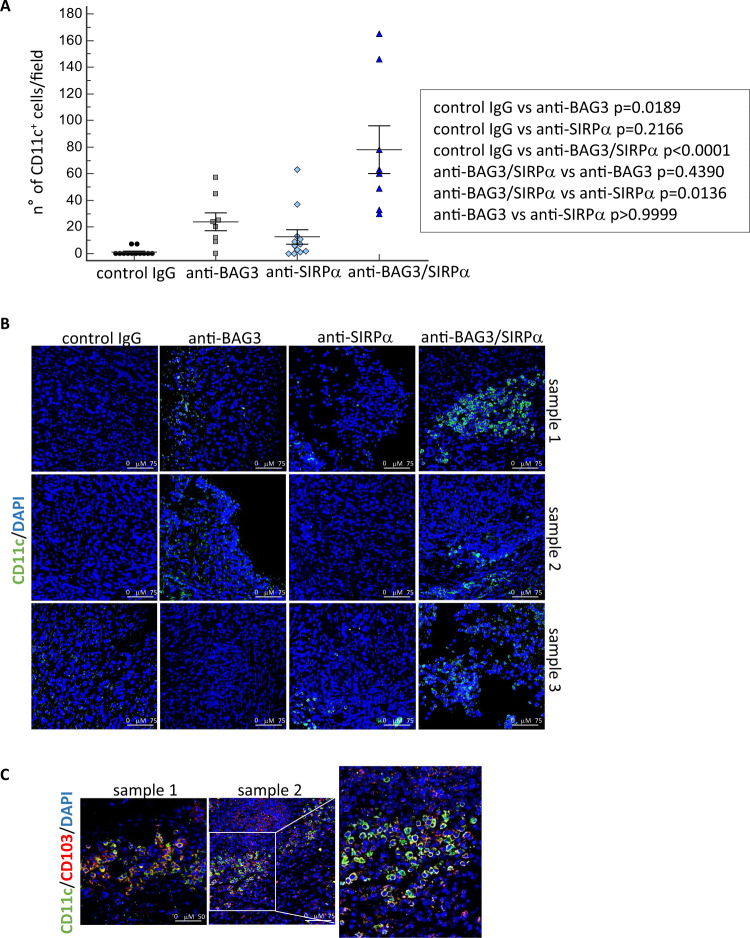
CD11c+ dendritic cells in mice tumors after antibodies treatments. **A** CD11c^+^ dendritic cells in excised tumors were revealed by confocal immunofluorescence microscopy with an anti-CD11c monoclonal antibody. Nuclei were counterstained with DAPI. Three to five fields, according to their size, of four tumors per group were examined. **B** Representative images of CD11c positivity. Images were acquired in sequential scan mode using the same acquisitions parameters (laser intensities, gain photomultipliers, pinhole aperture, objective×40, zoom 1) to compare treated samples and controls. Non-parametric Kruskal–Wallis test with Dunn’s correction was used for data analysis. **C** Expression of CD103 antigen (red) in CD11c (green)-positive cells.

**Fig. 5:**
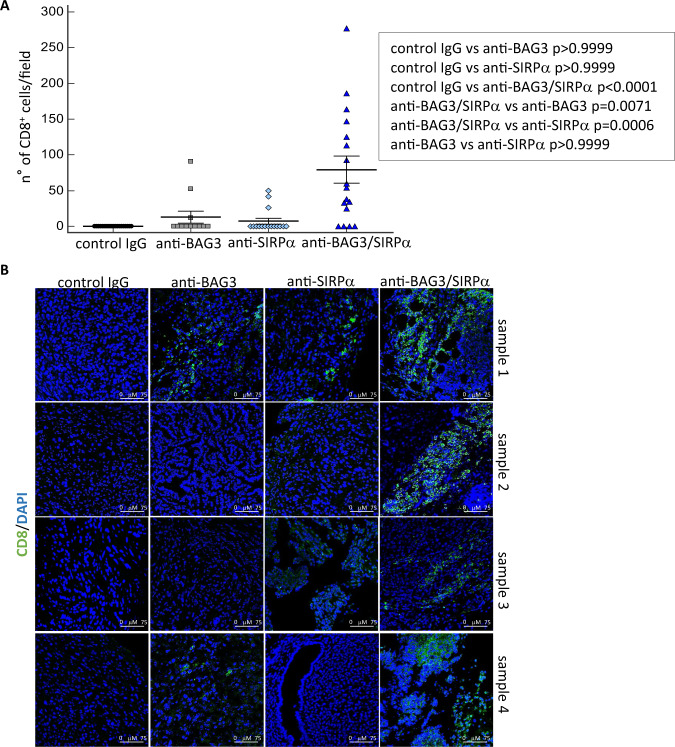
Effect of anti-SIRPα and anti-BAG3 antibodies on the recruitment of CD8^+^cells in tumors. **A** CD8^+^ lymphocytes were identified in excised tumors by immunofluorescence with an anti-CD8 monoclonal antibody in confocal microscopy. Nuclei were counterstained with DAPI. Three to seven fields, according to their size, of four tumors per group were examined and analyzed. Non-parametric Kruskal–Wallis test with Dunn’s correction was used for data analysis. **B** Representative images of CD8 positivity. Images were acquired in sequential scan mode, by using the same acquisitions parameters (laser intensities, gain photomultipliers, pinhole aperture, objective×40, zoom 1) to compare treated samples and controls.

We analyzed the effects of the treatments on the presence of dendritic cells and CD8^+^ T lymphocytes in tumor tissues. A modest increase in the number of CD11c^+^ cells was detected in allografts treated with each of the two antibodies, but a very higher increase was evident in mice treated with both antibodies (Fig. 4A, B). A more accurate analysis showed that CD11c^+^CD103^+^ dendritic cells represented a substantial part of the overall CD11c^+^ labeled cells (Fig. 4C). In good agreement with DC increase, also CD8^+^ lymphocytes, hardly detectable in untreated tumors, were observed in anti-BAG3- or anti-SIRPα- treated tumors and, at a very higher level, in tumors treated with both antibodies (Fig. 5A, B).

### CAFs activation and desmoplasia are abated in tumors of treated mice

**Fig. 6:**
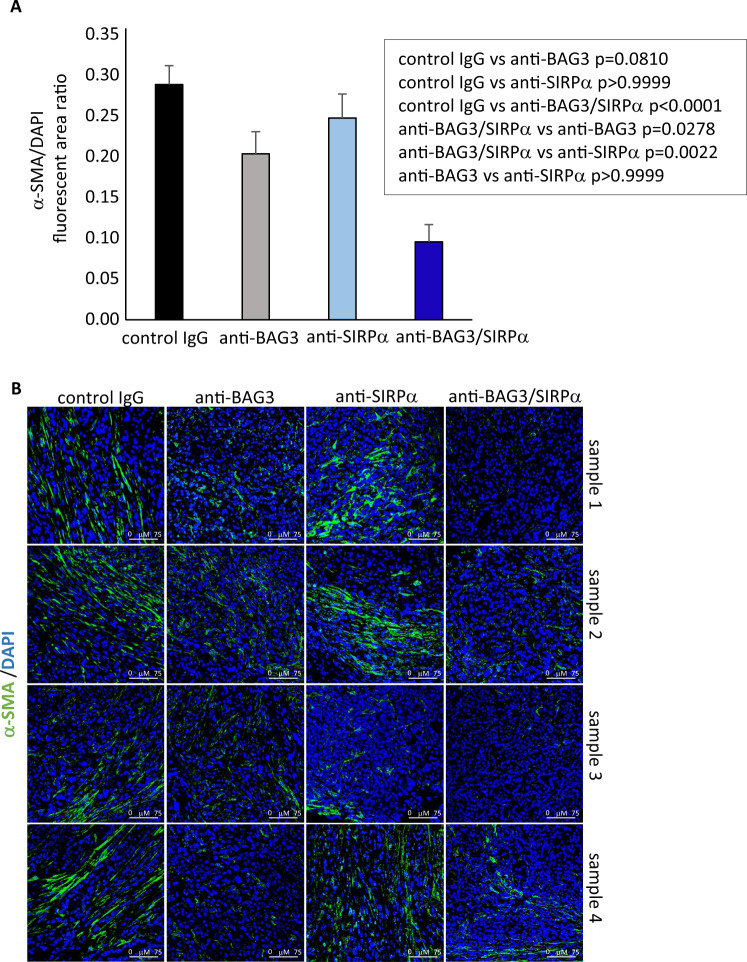
Analysis of CAF α-SMA expression in tumors from animals treated with the antibodies. **A** Relative fluorescence area of α-SMA-positive cells, calculated as ratio to DAPI staining using ImageJ software at 40 field magnification, in tumors excised from four animals of each group. For each tumor, two to ten fields, depending on their size, were analyzed. One-way ANOVA followed by Bonferroni’s post hoc test was used for data analysis. **B** Representative images.

**Fig. 7:**
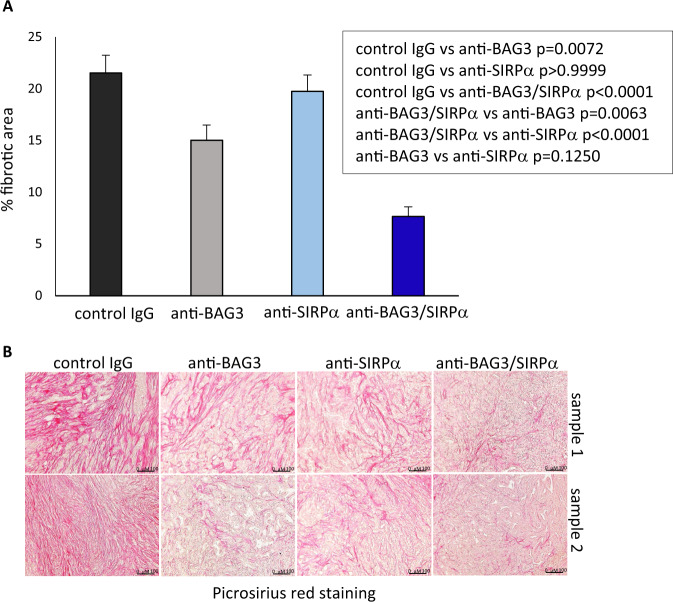
Tumor fibrosis in treated animals. **A** Collagen staining (Picrosirius red) and image analysis of tumor tissues (four animals per group). Fibrotic areas were analyzed and quantified as percent of whole areas in ≥10 different fields per sample. Non-parametric Kruskal–Wallis test with Dunn’s correction was used for data analysis. **B** Representative images.

We previously reported that treatment of pancreatic cancer heterotopic allografts with anti-BAG3 antibody down-modulated CAF activation and impaired the desmoplastic structure in pancreatic cancer stroma [13]. In agreement with results in heterotopic allografts, we observed a reduction of the expression of the activation marker α-SMA in CAFs. Such reduction was raised up to >70% (*p* < 0.0001) by co-treatment with the anti-SIRPα mAb, while treatment with the anti-SIRPα mAb alone did not result in any appreciable decrease (*p* > 0.999) of α-SMA positivity (Fig. 6). In parallel, fibrosis was impaired by about the 30% (0.007) and the 64% (*p* < 0.0001) by treatment with, respectively, anti-BAG3 and anti-BAG3 + anti-SIRPα mAbs, while the anti-SIRPα mAb did not significantly affect fibrosis when used alone (Fig. 7). Therefore, the effects of the combined blockade of the two pathways did not appear to simply reflect the sum of the effects of the single blockades, but instead SIRPα blockade, although unable by itself to modulate CAF activation and fibrosis, effectively contributed to the antifibrotic effect of the anti-BAG3 mAb.

## Discussion

In line with the currently pursued therapeutic strategies against pancreatic cancer [2–4, 7], our results support the concept that an action on more than one regulatory circuit in the tumor microenvironment can counteract neoplastic growth and metastatic process. For the design of such strategies, we need an in-depth knowledge of the interactions between the different tumor-microenvironment functional connections which, on the one hand, support tumor growth and, on the other, suppress the immune response. In this work, we addressed two regulatory pathways, one of which—BAG3/BAG3R—contributes to support the growth of pancreatic carcinoma through the pro-tumor activity of TAMs and CAFs stimulated by BAG3 [2, 3, 10–13], while the other—SIRPα/CD47—is an immune checkpoint that blocks the phagocytosis of neoplastic cells and, notably, the activation of dendritic cells [16–24]. The results indicate that the concerted blockade of the two pathways can lead to remarkable anti-tumor effects.

In the context of the molecules that regulate the interactions between pancreatic carcinoma and its microenvironment, BAG3 attracts interest for some characteristics: the ability to influence both TAMs and CAFs; its specific presence, as a secreted factor, in tumor tissues and not in normal ones; the lack of toxicity of anti-BAG3 antibodies in preclinical treatments, even in the long term [3, 10–15, 29]. The BAG3-governed pathway appears potentially a useful candidate in combination therapies. In this sense, it is noteworthy that an anti-BAG3 antibody is able to sensitize pancreatic carcinoma to the effect of an anti-PD-1 antibody [12]. The combined effect shown here on tumor growth and metastasization following the BAG3 and SIRPα blockade provides further evidence of the versatility of the anti-BAG3 tools in combination therapies.

A particularly interesting property of BAG3-blocking therapy is the destructive effect on desmoplasia [13]. Such property is relevant, given the importance of the desmoplastic arrangement of the stroma in supporting epithelial-mesenchymal transition, orchestrating the invasion of neoplastic cells, upsetting the anti-tumor immune response, and hampering tumor exposure to drugs [30–33]. Indeed, the development of a desmoplastic tumor microenvironment is a key element in pancreatic ductal adenocarcinoma carcinogenesis [30]. In the light of the importance of the stroma and the consequent role of CAFs on metastasization [34], it is not surprising the effect of BAG3 blockade, acting on CAFs [13], on the number of metastases per animal (Fig. 2A, B). Although BAG3 activity on CAFs—on which the desmoplastic implant mainly depends—is documented [13], it is necessary to define the CAF populations [35–44] involved and the mechanism leading to the impressive anti-fibrotic effect of BAG3 blockade. This topic is of great interest for the advancement of knowledge of the biology of pancreatic cancer and other fibrotic tumors, in which desmoplasia and mechanoreciprocity mechanisms play a fundamental role in resistance to therapies [45–48].

A remarkable observation that emerges from data analysis is that the effects of single and combined antibody treatments are distinct. Indeed, treatment with the anti-SIRPα antibody, while not having a significant effect per se on CAF activation and fibrosis, in these respects significantly contributed to the effect of the anti-BAG3 antibody (Figs. 4, 5). On the other hand, although SIRPα is known to block the activation and survival of dendritic cells, mainly through sequestration of the PI3K p85 subunit [17, 21], treatment with the anti-SIRPα antibody did not lead to an appreciable increase of dendritic cells in pancreatic tumor tissue, if not in combination with the anti-BAG3 antibody (Fig. 5). The increase in dendritic cells is a notable effect of the combined treatment, as these cells regulate the recruitment and activation of cytotoxic lymphocytes (which, in fact, were also increased in tumor tissue), and are crucial in the response to immunotherapies [49–52].

The experimental evidence shows that the effects of individual treatments are not as much impressive as the combo treatment with both antibodies. In fact, the results clearly point out a reciprocal influence of BAG3/BAG3R or SIRPα/CD47 blockade on the activity of the other pathway. This mutual influence is most likely due to the regulatory role that each pathway plays on the release of cytokines and chemokines in the tumor microenvironment, where several cellular components like CAFs, TAMs, MDSCs, and other myeloid cells, are involved in the complex biochemical crosstalk granting the tumor cells survival and proliferation. As shown in Fig. 3B, BAG3 and SIRPα blockade produces a noticeable effect on cytokines and chemokines clusters. The treatment with mAbs allowed to obtain useful information about the overall changes in the cytokines/chemokines assets, and a more accurate data mining could lead to a more precise identification of single elements of the clusters responsible of cancer fibrosis and of the recruitment of DCs and CD8^+^ lymphocytes.

In conclusion, our findings show that the blockades of BAG3/BAG3R and SIRPα/CD47 axes converge in eliciting a sound anti-tumor immune response against pancreatic cancer and in countering tumor growth and the metastatic process. These results highlight the functional integration of the two pathways in determining the global functional setting of the pancreatic cancer microenvironment and provide a proof of principle of the potential validity of a combined therapeutic treatment against BAG3 and SIRPα.

## Data Availability

The data that support the findings of this study are available from the corresponding author upon reasonable request.

## References

[1] Huang J, Lok V, Ngai CH, Zhang L, Yuan J, Lao XQ (2021). Worldwide burden of, risk factors for, and trends in pancreatic cancer. Gastroenterology.

[2] Balachandran VP, Beatty GL, Dougan SK (2019). Broadening the Impact of Immunotherapy to pancreatic cancer: Challenges and opportunities. Gastroenterology.

[3] Bear AS, Vonderheide RH, O’Hara MH (2020). Challenges and opportunities for pancreatic cancer immunotherapy. Cancer Cell.

[4] Gromisch C, Qadan M, Machado MA, Liu K, Colson Y, Grinstaff MW (2020). Pancreatic adenocarcinoma: Unconventional approaches for an unconventional disease. Cancer Res.

[5] Schizas D, Charalampakis N, Kole C, Economopoulou P, Koustas E, Gkotsis E (2020). Immunotherapy for pancreatic cancer: A 2020 update. Cancer Treat Rev.

[6] Hessmann E, Buchholz SM, Demir IE, Singh SK, Gress TM, Ellenrieder V (2020). Microenvironmental determinants of pancreatic cancer. Physiol Rev.

[7] Ho WJ, Jaffee EM, Zheng L (2020). The tumour microenvironment in pancreatic cancer—clinical challenges and opportunities. Nat Rev Clin Oncol.

[8] Leinwand J, Miller G (2020). Regulation and modulation of antitumor immunity in pancreatic cancer. Nat Immunol..

[9] Ammirante M, Rosati A, Arra C, Basile A, Falco A, Festa M (2010). IKK{gamma} protein is a target of BAG3 regulatory activity in human tumor growth. Proc Natl Acad Sci USA.

[10] Ray K (2015). Pancreatic cancer: New insights into PDAC growth promotion via a BAG3-mediated paracrine loop. Nat Rev Gastroenterol Hepatol.

[11] Rosati A, Basile A, D’Auria R, d’Avenia M, De Marco M, Falco A (2015). BAG3 promotes pancreatic ductal adenocarcinoma growth by activating stromal macrophages. Nat. Commun.

[12] Iorio V, Rosati A, D’Auria R, De Marco M, Marzullo L, Basile A (2018). Combined effect of anti-BAG3 and anti-PD-1 treatment on macrophage infiltrate, CD8^+^ T cell number and tumour growth in pancreatic cancer. Gut.

[13] Iorio V, De Marco M, Basile A, Eletto D, Capunzo M, Remondelli P (2019). CAF-derived IL6 and GM-CSF cooperate to induce M2-like TAMs-letter. Clin Cancer Res.

[14] Li C, An MX, Jiang JY, Yao HB, Li S, Yan J (2019). BAG3 suppresses loading of Ago2 to IL6 mRNA in pancreatic ductal adenocarcinoma. Front Oncol.

[15] De Marco M, Turco MC, Marzullo L (2020). BAG3 in tumor resistance to therapy. Trends Cancer.

[16] McCracken MN, Cha AC, Weissman IL (2015). Molecular pathways: Activating T cells after cancer cell phagocytosis from blockade of CD47 “don’t eat me” signals. Clin Cancer Res.

[17] Liu Q, Wen W, Tang L, Qin C-J, Qin CJ, Lin Y (2016). Inhibition of SIRPα in dendritic cells potentiates potent antitumor immunity. Oncoimmunology.

[18] Matlung HL, Szilagyi K, Barclay NA, van den Berg TK (2017). The CD47-SIRPα signaling axis as an innate immune checkpoint in cancer. Immunol Rev.

[19] Yanagita T, Murata Y, Tanaka D, Motegi SI, Arai E, Daniwijaya EW (2017). Anti-SIRPα antibodies as a potential new tool for cancer immunotherapy. JCI Insight.

[20] Gauttier V, Pengam S, Durand J, Biteau K, Mary C, Morello A (2020). Selective SIRPα blockade reverses tumor T-cell exclusion and overcomes cancer immunotherapy resistance. J Clin Invest.

[21] Kuo TC, Chen A, Harrabi O, Sockolosky JT, Zhang A, Sangalang E (2020). Targeting the myeloid checkpoint receptor SIRPα potentiates innate and adaptive immune responses to promote anti-tumor activity. J Hematol Oncol.

[22] Logtenberg MEW, Scheeren FA, Schumacher TN (2020). The CD47-SIRPα immune checkpoint. Immunity.

[23] Liu J, Xavy S, Mihardja S, Chen S, Sompalli K, Feng D (2020). Targeting macrophage checkpoint inhibitor SIRPα for anticancer therapy. JCI Insight.

[24] Zhang W, Huang Q, Xiao W, Zhao Y, Pi J, Xu H (2020). Advances in anti-tumor treatments targeting the CD47/SIRPα axis. Front Immunol.

[25] Behl C (2016). Breaking BAG: The co-chaperone BAG3 in health and disease. Trends Pharm Sci.

[26] Verjan Garcia N, Umemoto E, Saito Y, Yamasaki M, Hata E, Matozaki T (2011). SIRPα/CD172a regulates eosinophil homeostasis. J Immunol.

[27] Boj SF, Hwang CI, Baker LA, Chio II, Engle DD, Corbo V (2015). Organoid models of human and mouse ductal pancreatic cancer. Cell.

[28] Beatson R, Sproviero D, Maher J, Wilkie S, Taylor-Papadimitriou J, Burchell JM (2011). Transforming growth factor-β1 is constitutively secreted by Chinese hamster ovary cells and is functional in human cells. Biotechnol Bioeng.

[29] Basile A, De Marco M, Festa M, Falco A, Iorio V, Guerriero L (2019). Development of an anti-BAG3 humanized antibody for treatment of pancreatic cancer. Mol Oncol.

[30] Storz P, Crawford HC (2020). Carcinogenesis of pancreatic ductal adenocarcinoma. Gastroenterology.

[31] Bulle A, Lim KH (2020). Beyond just a tight fortress: Contribution of stroma to epithelial-mesenchymal transition in pancreatic cancer. Signal Transduct Target Ther.

[32] Yu S, Zhang C, Xie KP (2021). Therapeutic resistance of pancreatic cancer: Roadmap to its reversal. Biochim Biophys Acta Rev Cancer.

[33] Peran I, Dakshanamurthy S, McCoy MD, Mavropoulos A, Allo B, Sebastian A (2021). Cadherin 11 promotes immunosuppression and extracellular matrix deposition to support growth of pancreatic tumors and resistance to gemcitabine in mice. Gastroenterology.

[34] Crawford HC, Pasca di Magliano M, Banerjee S (2019). Signaling networks that control cellular plasticity in pancreatic tumorigenesis, progression, and metastasis. Gastroenterology.

[35] Öhlund D, Handly-Santana A, Biffi G, Elyada E, Almeida AS, Ponz-Sarvise M (2017). Distinct populations of inflammatory fibroblasts and myofibroblasts in pancreatic cancer. J Exp Med.

[36] Belle JI, DeNardo DG (2019). A single-cell window into pancreas cancer fibroblast heterogeneity. Cancer Discov.

[37] Biffi G, Oni TE, Spielman B, Hao Y, Elyada E, Park Y (2019). IL1-induced JAK/ STAT signaling is antagonized by TGFbeta to shape CAF heterogeneity in pancreatic ductal adenocarcinoma. Cancer Discov.

[38] Elyada E, Bolisetty M, Laise P, Flynn WF, Courtois ET, Burkhart RA (2019). Cross-species single-cell analysis of pancreatic ductal adenocarcinoma reveals antigen-presenting cancer-associated fibroblasts. Cancer Discov.

[39] Vennin C, Mélénec P, Rouet R, Nobis M, Cazet AS, Murphy KJ (2019). CAF hierarchy driven by pancreatic cancer cell p53-status creates a pro-metastatic and chemoresistant environment via perlecan. Nat Commun.

[40] Garcia PE, Adoumie M, Kim EC, Zhang Y, Scales MK, El-Tawil YS (2020). Differential contribution of pancreatic fibroblast subsets to the pancreatic cancer stroma. Cell Mol Gastroenterol Hepatol.

[41] Helms E, Onate MK, Sherman MH (2020). Fibroblast heterogeneity in the pancreatic tumor microenvironment. Cancer Discov.

[42] Sahai E, Astsaturov I, Cukierman E, DeNardo DG, Egeblad M, Evans RM (2020). A framework for advancing our understanding of cancer-associated fibroblasts. Nat Rev Cancer.

[43] Steele NG, Biffi G, Kemp SB, Zhang Y, Drouillard D, Syu L (2021). Inhibition of hedgehog signaling alters fibroblast composition in pancreatic cancer. Clin Cancer Res.

[44] Feldmann K, Maurer C, Peschke K, Teller S, Schuck K, Steiger K (2021). Mesenchymal plasticity regulated by Prrx1 drives aggressive pancreatic cancer biology. Gastroenterology.

[45] Piersma B, Hayward MK, Weaver VM (2020). Fibrosis and cancer: A strained relationship. Biochim Biophys Acta Rev Cancer.

[46] Shalapour S, Lin XJ, Bastian IN, Brain J, Burt AD, Aksenov AA (2017). Inflammation-induced IgA^+^ cells dismantle anti-liver cancer immunity. Nature.

[47] Mariathasan S, Turley SJ, Nickles D, Castiglioni A, Yuen K, Wang Y (2018). TGFβ attenuates tumour response to PD-L1 blockade by contributing to exclusion of T cells. Nature.

[48] Boulter L, Bullock E, Mabruk Z, Brunton VG (2021). The fibrotic and immune microenvironments as targetable drivers of metastasis. Br J Cancer.

[49] Böttcher JP, Reis e Sousa C (2018). The role of type 1 conventional dendritic cells in cancer immunity. Trends Cancer.

[50] Williford JM, Ishihara J, Ishihara A, Mansurov A, Hosseinchi P, Marchell TM (2019). Recruitment of CD103^+^ dendritic cells via tumor-targeted chemokine delivery enhances efficacy of checkpoint inhibitor immunotherapy. Sci Adv.

[51] Ferris ST, Durai V, Wu R, Theisen DJ, Ward JP, Bern MD (2020). cDC1 prime and are licensed by CD4^+^ T cells to induce anti-tumour immunity. Nature.

[52] Mayoux M, Roller A, Pulko V, Sammicheli S, Chen S, Sum E (2020). Dendritic cells dictate responses to PD-L1 blockade cancer immunotherapy. Sci Transl Med.

